# Contributions to Theoretical Dentistry

**Published:** 1858-07

**Authors:** Ad. Zur Nedden

**Affiliations:** Zahnarzt in Nurnberg.


### ARTICLE III.

*Contributions to Theoretical Dentistry.* By Ad. Zur Nedden, Zahnarzt in Nurnberg. Translated from der Zahnarzt, for Am. Jour. Dent. Science.

*All parts of the living organism are composed of cellules, or, at least, the very origin of cellular forms may be more or less distinctly deduced.*

The teeth being parts of the organism, are wholly implicated in this dogma, the importance of which has only began to be recognized since the nature, origin, progressive development, and the composition of the teeth have been cleared up by microscopic anatomy and chemistry. In all preceding views about this subject, therefore, we do not find it taken as much into consideration as its knowledge deserves for scientific appreciation of dentistry.

As we are about giving proofs to this assertion, we refer to the following facts:

The inorganic parts of the teeth consisting almost entirely of phosphate and carbonate of calcium, are imbedded in a cartilaginous-like substance, resulting into lime by undergoing the act of boiling, which may be obtained whenever the calcareous salts become dissolved by operating upon them with diluted hydrochloric acid, and then it shows the same generally fibrinous structure as the tooth does, being still intact. These fibrinous elements, by boiling with water, may not only be separated, but even the isolation of some of the roundish granular cellules, with their distinct contours, may be effected, which undoubtedly claim for themselves the same importance in regard to the teeth, as the bony particles in the bones or the cartilaginous cellules in the cartilages, because they are in all probability the same formation as the interglobular spaces (interglobularraume) designated by Czermak.*

Furthermore, the first origin of the teeth presents itself in the shape of very delicate bubbles, "like being the ground and soil wherefrom the future teeth become formed and synonymous with the cystoblastema, the beginning of all tissues." (Franko.) It is in these bubbles that the gelatinous substance develops itself out of the granular cellular tooth-germ, previous to the deposit of calcareous salts, beginning for the temporary teeth from the 5th till 6th month of embryonal life; for the permanent ones during childhood.

We thus notice a process, which as regards both its origin and development, harmonizes generally with that of the formation of all tissues, but in many points in particular with that of bony tissues, as in the latter too a germ bed, callous or cartilaginous formation and afterwards a deposit of calcareous salts is to be found. It therefore cannot be denied that the teeth raise up from cellular foundation just

*Zeitschriff f. wissensch. Zoologie, bd. 2, p. 295. In the isolation of those fibrils and cellules, I always effected after the manner of *Hoppe*. Arch. f. Path. Anatom. bd. 5, p. 170.

in the same way as observed in all other tissues. For this reason also, all the doctrines must be applied to the teeth, delivered by Virchow in his article on cellular pathology.* They not only form the basis of a right view about all physiologic and pathologic facts, but also of rational therapeutics.

The teeth now, as well as all other parts of the body, are by means of the blood and nerves, in the strictest reciprocal dependency. Blood and nerves are indeed the centralizing points, without whose integrity the cellules, the real factors of all living functions, would not derive the necessary supply of their nutrition. (Virchow.) Any disturbances in the functiôns of the nervous system, the circulation of the blood or changes in the latter composition, whether they are primary symptoms or rather consequences of some local affections in other parts, result in the same way into affections of the teeth, because they are, under all circumstances; also disturbances of cellular activity, cellular life, change of matter and nutrition. Such nutritive changes may go on in all living elements, and for the same reason, of course, in the teeth, but the influences are always external ones, never arising in the elements themselves.

Whatever might be said as to the not always existing possibility to point out the aforesaid nutritive changes by scientific investigation, yet by the progressive discoveries in pathological anatomy, we are compelled to believe that in all processes known as diseases, material variations of the elements are really at the bottom of changed nutrition; though we do not mean to say that the material changes of form and mixture of the tissues form, in all cases, the most important part of diseases. On the contrary, they may be co-ordinate or subordinate to the other concomitant symptoms. For we know not only of very grave changes of important organs without their functions being deranged at all, but also of uncommon disturbances of them when still

*Arch. f. Path. Anat. bd. 8, heft. 1.

no great changes are hereby observed. In the same way, too, diseases of the teeth come very often to our knowledge, where it is either quite easy to point out the structural changes which have taken place, and still they do not result in other than very insignificant symptoms, or we come across cases known as affections accompanied by the most excruciating sufferings, which are almost unmanageable by any treatment, because they baffle even every attempt of anatomical investigation.

As to the different tissues of the body, we observe various gradations of vital activity, as well as various degrees of height, as it regards the corresponding sensibility of the latter. (Virchow.) The teeth and bones, reversedly to the nerves occupying the first rank, are on the lowest degree, with which corresponds the distribution of blood in the teeth being quite peculiar, as there are no vessels intruding between the smallest particles of tooth-substance. In spite of that, it is very probable that fluids circulate in the fine canals of the tooth by means of the capillary blood circulation, carrying the necessary supply for its nutrition. The artery entering the tooth cavity, is of a very small size in proportion with the depending tissues, in particular when compared with the conditions of other parts. The capillary plexus on the other hand is very voluminous and developed in such a manner, that the blood entering the tooth cavity effects its distribution and circulation but very slowly. The nutritive fluid, therefore, absorbed by exosmosis, is fully in want of a power regulating the circulation, absorption or however it will be called, in the tooth canals like that going on in other tissues. Of course, it results after all, in a less active change of matter, and may be found existing in proportion to the smaller quantity of organic substances beside the calcareous salts. Under such circumstances, we cannot be astonished at the fact of the slow development, progress and disappearance of disturbances relating to the nutritive elements of the teeth. It is hereby quite evident to the every day experience that the regulating activities cannot carry out their effects otherwise than very slowly, and are, therefore, for the most part, without any appreciable results.

The branches of the fifth pair of nerves supplying the teeth and their adnexa, consist entirely of sensitive fibrils; but, at the same time with the artery, a nervous cord enters into each tooth cavity, radiating beside the capillary plexus. Both these small organs in common with some adhesive tissue and a very thin fibrinous cuticle, occupies the pulp cavity. Any influences experienced upon the sensibility of the tooth nerve inside of the tooth cavity, whether they are depressing or exciting, can only show their effects by means of the blood or the nerve, if we take out possible local influences in the tooth cavity. For this reason, morbid changes in the tooth substance will never manifest themselves by pain, as long as they go no further and do not implicate other parts supplied with nerves.

It is, however, quite different if the bones with the periosteum and the soft parts of the tooth system are the location of pathologic changes. As far then as it regards the nutritive alterations of the bones and the fibrinous periosteum, we observe among their accompanying symptoms more or less pain, though they are not in all cases to be taken for excited sensibility along the tooth branches of the fifth pair, but are usually regarded by people as toothache, according as they come to the dentist's notice.

In all parts of the tooth system, pulp, periosteum of the root, gum and even nerves, the pathologic changes may be said to be almost in every instance followed by pain, the severity of which depends undoubtedly upon the local conditions, and that for the reason of the peripheric radiation of the tooth nerves inside of the very vascular pulp; there enclosed by rocky walls, they have to progress in bony canals beside blood carrying vessels, while their extension into the periosteum of the root between the strong walls of the alveoli and the roots of the teeth encounters much difficulty. If furthermore, we bear in mind the fibrinous structure of the gum, we cannot be surprised by alterations on the nervous functions, if even they are the results of very insignificant disturbances in the above named parts, which do not seem to be in proportion with the originating causes. Indeed, there are but a few organs wherein we observe anything of this kind. In taking them into consideration, however, the physiological nature of the fifth pair ought not to be overlooked, but it is to the above named local conditions we have to reduce not a small amount, as to the causes of such degrees of pain most commonly found in these affections.

Besides that, we know still many other affections in distant organs, particularly of the central nervous system, the digestive apparatus and the female organs of generation, which by means of reflective action, give rise to changes in the fifth pair of nerves; they always come under observation in the shape of painful aches, the duration, progress and severity of which are dependent upon the peculiar circumstances of each case.

We, therefore, dare to pronounce toothache on the one hand, as a symptom of all pathologic process in the tooth system, with the exception of those in the tooth substance itself, whenever they are confined to the latter, and no other nerves carrying parts implicated with them; on the other hand, as a reflective symptom caused by affections of other distant organs. The designation "idiopathic toothache," met with in almost every treatise, will merely have it understood, that the apprehensible or suspected material basis is to be found nowhere else than in the tissue of the nerve, the latter alone being the affected part. This kind of toothache, indeed, is as numerous in relation to the character and symptoms, that there seems to be no other possibility and chance to fix them than by corresponding the toothache as a nervous local affection. In most cases, however, minute investigations, based upon scientific principles, will enable us to trace the causes into the dental tissue or other parts, and then the existence of idiopathic toothache must be reduced to very few cases, at any rate not quite as many as is generally presumed, because the pure hyperesthesies of the fifth pair originating in a nervous change itself, not being secondary symptoms, belong to diseases of rare occurrence.

